# An orphan kinesin controls trypanosome morphology transitions by targeting FLAM3 to the flagellum

**DOI:** 10.1371/journal.ppat.1007101

**Published:** 2018-05-29

**Authors:** Tai An, Ziyin Li

**Affiliations:** Department of Microbiology and Molecular Genetics, McGovern Medical School, University of Texas Health Science Center at Houston, Houston, TX, United States of America; University of California, Los Angeles, UNITED STATES

## Abstract

*Trypanosoma brucei* undergoes life cycle form transitions from trypomastigotes to epimastigotes in the insect vector by re-positioning the mitochondrial genome and re-locating the flagellum and flagellum-associated cytoskeletal structures. The mechanism underlying these dramatic morphology transitions remains poorly understood. Here we report the regulatory role of the orphan kinesin KIN-E in controlling trypanosome morphology transitions. KIN-E localizes to the flagellum and is enriched at the flagellar tip, and this localization depends on the C-terminal m-calpain domain III-like domains. Depletion of KIN-E in the trypomastigote form of *T*. *brucei* causes major morphology changes and a gradual increase in the level of EP procyclin, generating epimastigote-like cells. Mechanistically, through its C-terminal importin α-like domain, KIN-E targets FLAM3, a flagellar protein involved in morphology transitions, to the flagellum to promote elongation of the flagellum attachment zone and positioning of the flagellum and flagellum-associated cytoskeletal structure, thereby maintaining trypomastigote cell morphology. Our findings suggest that morphology transitions in trypanosomes require KIN-E-mediated transport of FLAM3 to the flagellum.

## Author summary

*Trypanosoma brucei*, the causative agent of sleeping sickness in humans and nagana in cattle in sub-Saharan Africa, has a complex life cycle by alternating between the tsetse fly vector and the mammalian hosts. In the gut of tsetse flies, trypanosomes undergo life cycle transitions from the trypomastigote form to the epimastigote form by re-positioning the mitochondrial genome and re-locating the flagellum and flagellum-associated cytoskeletal structures. Previous work demonstrated that elongation of the flagellum attachment zone plays an important role in controlling morphology transitions, but how it is regulated remains poorly understood. This work discovered that an orphan kinesin plays an essential role in regulating trypanosome morphology transitions. This novel kinesin localizes to the flagellum and targets FLAM3, one of the two flagellar proteins involved in morphology transitions, to the flagellum. This work suggests that trypanosome morphology transitions require kinesin-mediated transport of FLAM3 to the flagellum to promote the elongation of the flagellum attachment zone, thereby maintaining flagellum-cell body attachment and positioning the flagellum and flagellum-associated cytoskeletal structures to assume trypomastigote cell morphology.

## Introduction

Trypanosomatids, a group of protozoan parasites consisting of *Trypanosoma brucei*, *Trypanosoma cruzi*, and *Leishmania spp*., transition to different developmental forms with distinct cell morphology during their life cycle between the insect vectors and the mammalian hosts. These life cycle forms are distinguished by the relative position of the kinetoplast, the cell’s mitochondrial genome, to the nucleus, the cellular location from which the flagellum emerges, and the length of the free, unattached flagellum [1]. Inside the proventriculus of the tsetse fly vector, *T*. *brucei* differentiates from trypomastigote form to epimastigote form, which then undergoes an asymmetrical cell division and further develops to metacyclic form, the mammal-infective form of the parasite, in the salivary gland [1]. Although the molecular mechanisms underlying the transitions between these life cycle forms in trypanosomatids remain poorly understood, several proteins, including some RNA-binding proteins and a few flagellum-associated cytoskeletal proteins, were recently found to be involved in life cycle transitions in *T*. *brucei* [2,3,4,5,6,7]. The involvement of RNA-binding proteins ALBA3/4 [3] and RBP6 [2] in trypanosome life cycle transitions suggests a posttranscriptional regulation scheme, but mechanistically how these proteins contribute to this process is still elusive. The involvement of two flagellum attachment zone (FAZ) proteins in the flagellum, ClpGM6 and FLAM3 [4,5], and two intracellular FAZ proteins, FAZ9 [6] and TbSAS-4 [7], in life cycle form transitions suggests that the morphology transitions require the modulation of flagellum-associated cytoskeletal structures mediated by these FAZ proteins.

Kinesins are evolutionarily conserved microtubule-based motor proteins performing crucial roles in regulating microtubule dynamics and intracellular transport [8]. *T*. *brucei* possesses an expanded repertoire of kinesin-like proteins, including 13 kinetoplastid-specific kinesins and 15 orphan kinesins, most of which are of unknown function [9]. Previous work on Aurora B kinase-associated proteins identified two orphan kinesins, KIN-A and KIN-B, as nucleus- and spindle-associated kinesin proteins required for spindle assembly and chromosome segregation in *T*. *brucei* [10]. Given the essential roles of KIN-A and KIN-B in mitosis, they may function to compensate for the absence of mitotic kinesin homologs, such as the spindle motor protein BimC, the central spindle kinesin MKLP1/Pavarotti/ZEN-4, or the chromokinesin KLP3A, in *T*. *brucei*. Other studies uncovered the requirement of two kinetoplastid-specific kinesins, KIN-C and KIN-D, for maintaining cell morphology by modulating the organization of the subpellicular microtubule corset [11,12,13]. These findings highlighted the diverse cellular functions of kinetoplastid-specific kinesins and orphan kinesins, and further motivated us to explore the function of other kinesins in *T*. *brucei*.

To understand the potential roles of other orphan kinesins in *T*. *brucei*, their subcellular localization and biological functions were investigated in the procyclic form of *T*. *brucei*. Here we report one of the orphan kinesins, which is encoded by Tb927.5.2410 and was named KIN-E, and its essential function in targeting FLAM3, a FAZ flagellum domain protein crucial for trypanosome morphology transitions [4,14], to the flagellum to promote FAZ elongation and organelle positioning, thus maintaining flagellum-cell body attachment. These findings identify a new regulator and its mechanistic role in controlling trypanosome life cycle form transitions.

## Results

### KIN-E localizes to the flagellum and is enriched at the flagellar tip

**Fig 1:**
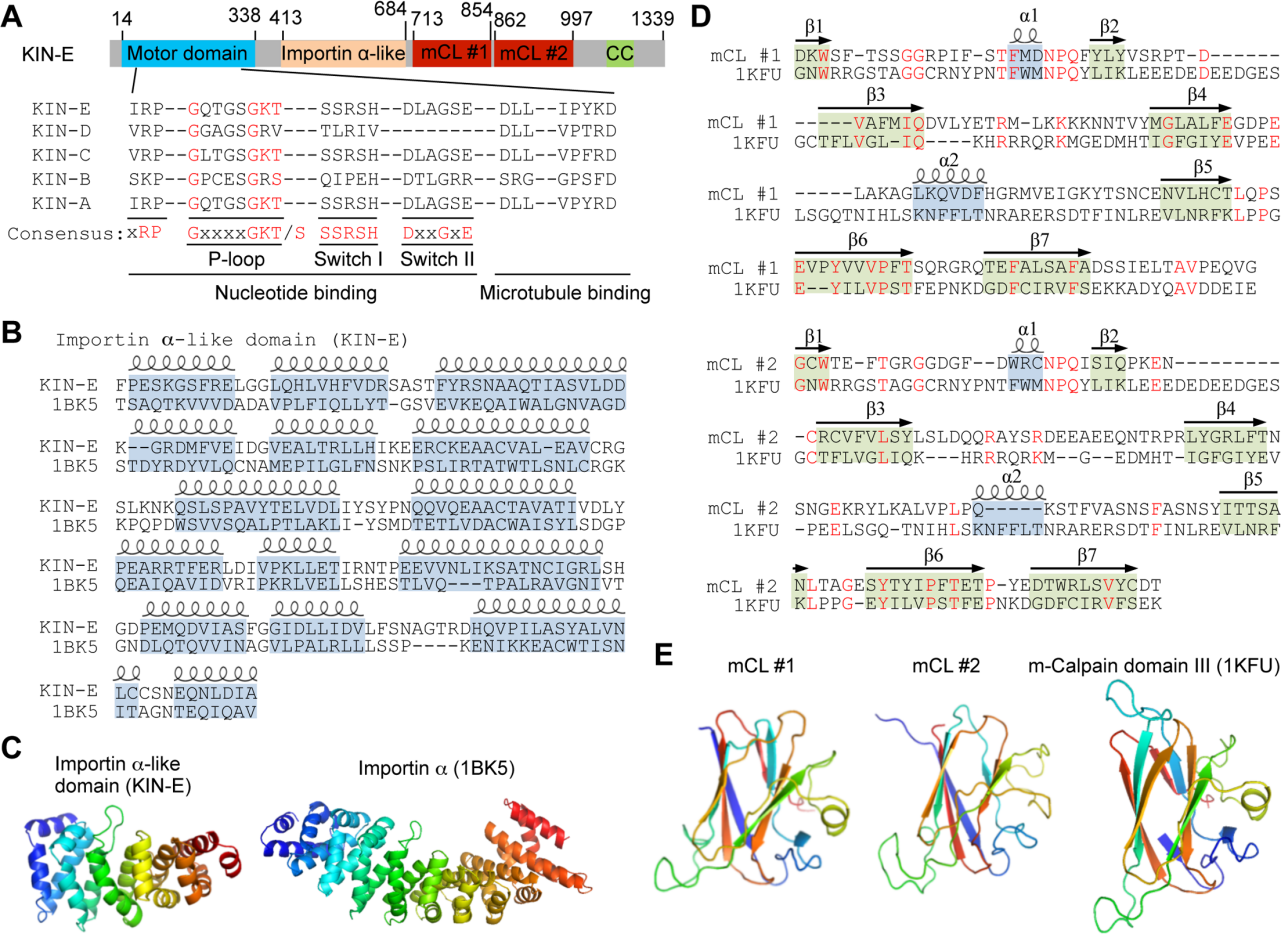
Analysis of the structural motifs in KIN-E, an orphan kinesin in *T*. *brucei*. **(A).** Schematic illustration of the structural motifs in KIN-E. The putative nucleotide-binding motif and the putative microtubule-binding motif of the kinesin motor domain of KIN-E were aligned with that of other orphan kinesins reported previously. The conserved glycine, lysine and threonine/serine residues in the P-loop of the nucleotide-binding motif were highlighted in red. mCL, m-Calpain domain III-like domain. CC, coiled coil. (**B**). Alignment of the putative importin α-like domain in KIN-E with the importin α protein from *Saccharomyces cerevisiae* (PBD code: 1BK5). The α-helical structures were indicated at the top of the aligned sequences. (**C**). Homology modeling of the importin α-like domain in KIN-E, using the *S*. *cerevisiae* importin α protein (PBD code: 1BK5) as the template. Note that the importin α-like domain in KIN-E is only about half size of the *S*. *cerevisiae* importin α protein. (**D**). Alignment of the m-calpain domain III-like domains (mCL#1 and mCL#2) of KIN-E with the domain III of the human m-calpain protein (PBD code: 1KFU). The α-helix structures and the β-sheet structures were indicated at the top of the aligned sequences. (**E**). Homology modeling of the m-calpain domain III-like domains in KIN-E, using the human m-calpain domain III (PBD code: 1KFU) as the template.

In an attempt to understand the function of the orphan kinesins in *T*. *brucei*, we first determined the subcellular localization of the remaining 13 orphan kinesins, each of which was tagged with a triple HA epitope at their respective endogenous locus, by immunofluorescence microscopy. One of these orphan kinesins, which is encoded by Tb927.5.2410, was found to localize to the flagellum and was enriched at the flagellar tip, and thus was further characterized. We named it KIN-E, following previous nomenclature of the four kinetoplastid-specific kinesins and orphan kinesins (KIN-A to KIN-D) [10,11,12,13]. KIN-E contains an N-terminal kinesin motor domain (MD), which comprises a conserved nucleotide-binding domain, consisting of a highly conserved P-loop (phosphate-binding loop) motif and two conserved switch motifs (switch I and switch II), and a conserved microtubule-binding motif (Fig 1A). Swiss modeling [15] showed that KIN-E contains an unusual importin α-like domain, which is about half size of the yeast importin α protein (Fig 1A–1C), and two m-calpain domain III-like domains, which is characterized by an antiparallel β-sandwich of a pair of four β-sheets [16] (Fig 1A, 1D and 1E), in addition to a small coiled-coil motif at the C-terminus (Fig 1A). The two m-calpain domain III-like domains (abbreviated as mCL#1 and mCL#2) in KIN-E share similar folds with the domain III of the human m-calpain (Fig 1D and 1E), suggesting that mCL#1 and mCL#2 in KIN-E may possess similar functions. KIN-E appears to be different from many known kinesins that contain mostly coiled-coil motifs at their C-terminus [17].

**Fig 2:**
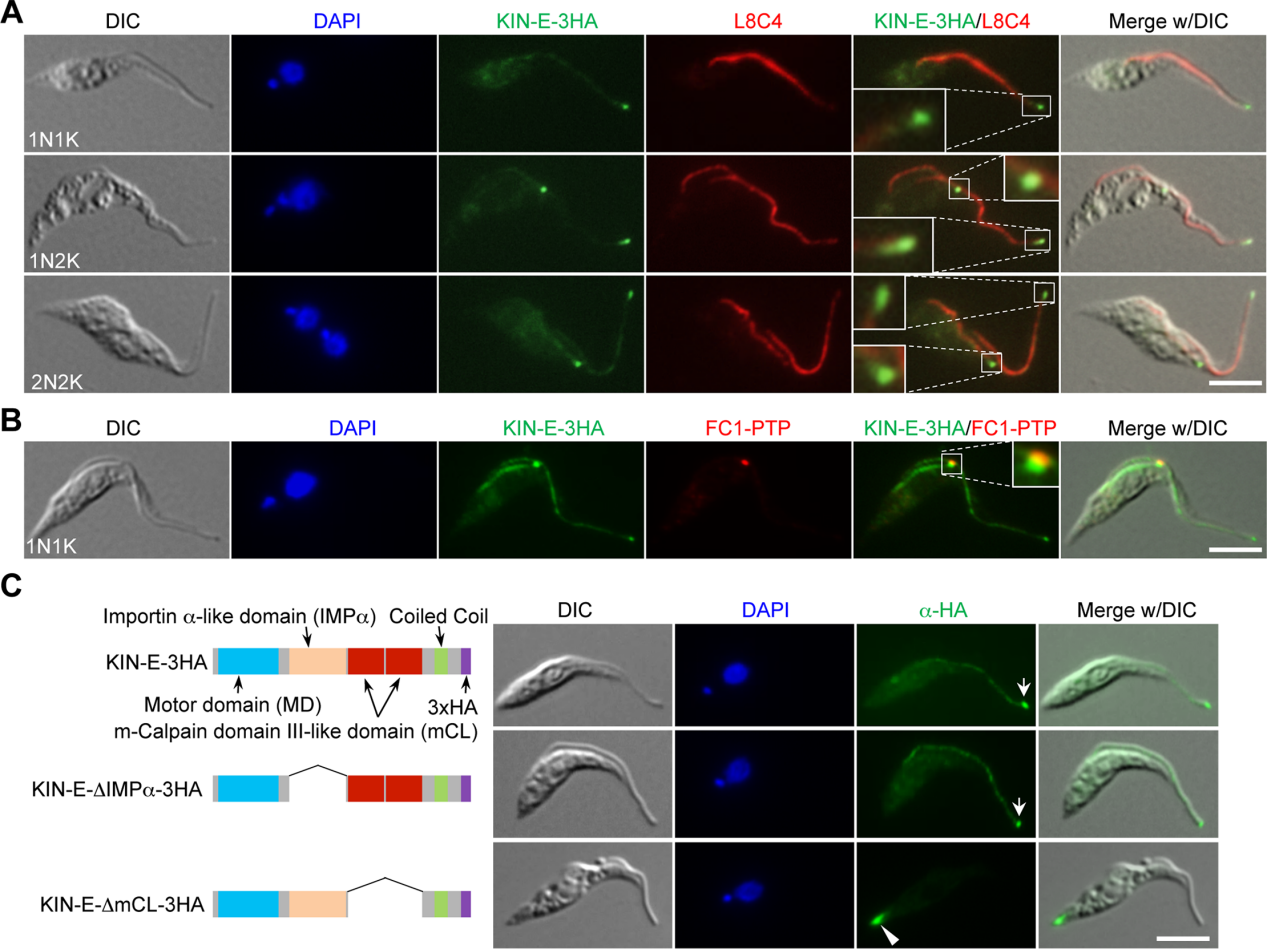
KIN-E localizes to the flagellum and is enriched at the flagellar tip. (**A**). KIN-E localizes to the distal tip of the flagellum. KIN-E was endogenously tagged with a triple HA epitope, and detected with FITC-conjugated anti-HA antibody. Cells were co-immunostained with anti-PFR2 antibody (L8C4), and counterstained with DAPI for nuclear and kinetoplast DNA. Scale bar: 5 μm. (**B**). KIN-E localizes to the flagella connector region. Cells co-expressing endogenously 3HA-tagged KIN-E and PTP-tagged FC1 were co-immunostained with FITC-conjugated anti-HA monoclonal antibody and anti-Protein A polyclonal antibody, and counterstained with DAPI for DNA. Scale bar: 5 μm. (**C**). Subcellular localization of ectopically expressed KIN-E and its various mutants in the 29–13 cell line. Wild-type KIN-E, the importin α-like domain deletion mutant (KIN-E-ΔIMPα), and the m-calpain domain III (mCL#1& mCL#2) deletion mutant (KIN-E-ΔmCL) were each tagged with a triple HA epitope at the C-terminus and ectopically expressed in the 29–13 cell line. Cells were incubated with 1.0 μg/ml tetracycline for 24 h and immunostained with FITC-conjugated anti-HA mAb. Arrows indicate the enrichment of KIN-E and KIN-E-ΔIMPα at the flagellar tip, and the arrowhead indicates KIN-E-ΔmCL at the posterior tip. Scale bar: 5 μm.

The subcellular localization of KIN-E during the cell cycle of *T*. *brucei* was investigated by immunofluorescence microscopy. Endogenously 3HA-tagged KIN-E is enriched at the distal tips of both the new and old flagella throughout the cell cycle and also localizes along the entire length of the flagella at a lower level (Fig 2A). At the distal tip of the new flagellum, KIN-E partly overlaps with the flagella connector protein FC1 [6] (Fig 2B). To investigate the potential contribution of the importin α-like domain and the two m-calpain domain III-like domains to KIN-E localization, we ectopically expressed KIN-E mutants deleted of the importin α-like domain (KIN-E-ΔIMPα) or the two m-calpain domain III-like domains (KIN-E-ΔmCL) in the 29–13 cell line, and then examined the subcellular localization of these mutants by immunofluorescence microscopy. The KIN-E-ΔIMPα mutant, which lacks the importin α-like domain, is still localized to the flagellum and is enriched at the flagellar tip (Fig 2C, arrow), similar to the wild-type KIN-E (Fig 2C, arrow), suggesting that the importin α-like domain is not required for KIN-E localization. Intriguingly, the KIN-E-ΔmCL mutant is not localized at the flagellum and the flagellar tip, but instead at the posterior end of the cells (Fig 2C, arrowhead), indicating that the m-calpain domain III-like domains in KIN-E are required for targeting KIN-E to the flagellum. Given that kinesins are microtubule plus end-directed motor proteins, it is likely that the KIN-E-ΔmCL mutant is directed to the cell posterior, the plus ends of the cytoskeletal subpellicular microtubules in *T*. *brucei*.

### Depletion of KIN-E causes major morphology changes and an increase in the level of EP procyclin

**Fig 3:**
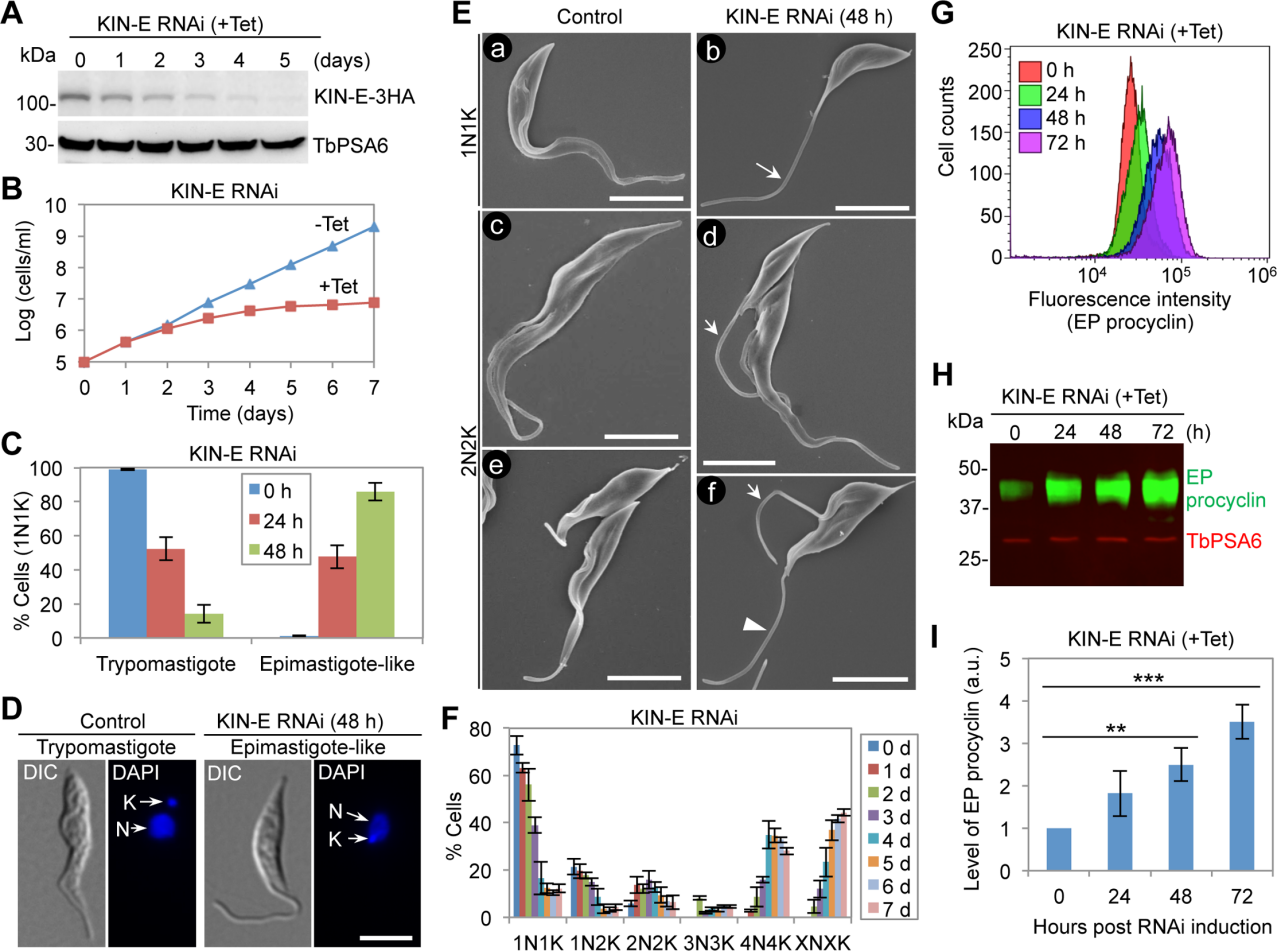
RNAi-mediated ablation of KIN-E caused a dramatic change of cell morphology. (**A**). Western blotting to monitor the efficiency of RNAi against KIN-E. KIN-E was endogenously tagged with a triple HA epitope in cells containing the KIN-E RNAi construct. TbKIN-E-3HA was detected by anti-HA antibody. TbPSA6, the alpha6 subunit of the 26S proteasome, served as the loading control. (**B**). Effect of KIN-E RNAi on cell proliferation. (**C**). Percentages of 1N1K cells of trypomastigote and epimastigote-like morphology in control and KIN-E RNAi cells. Cell morphology was determined based on the position of the kinetoplast relative to the nucleus as shown in panel D below. A total of 100 1N1K cells were counted for each time point. Error bars indicated S.D. from three independent experiments. (**D**). Cell morphology of control and KIN-E RNAi cells examined under a light microscope. N, nucleus; K, kinetoplast DNA. Scale bar: 5 μm. (**E**). Cell morphology of control and KIN-E RNAi cells examined by scanning electron microscopy. Shown in panels a, c, and e are control cells, whereas panels b, d, and f are KIN-E RNAi cells. The arrow in panel b indicates the long, unattached flagellum. Arrows in panels d and f indicate the long, unattached new flagellum, and the arrowhead in panel f indicates the long, unattached old flagellum. Scale bars: 5 μm. (**F**). Effect of KIN-E RNAi on cell cycle progression examined by quantitation of cells with different numbers of nucleus (N) and kinetoplast DNA (K). A total of 120 cells were counted for each time point. Error bars indicated S.D. from three independent experiments. (**G**). Flow cytometry analysis of EP procyclin expression in control and KIN-E RNAi cells. Cells were immunostained with anti-EP procyclin and FITC-conjugated mouse IgG, and then analyzed by flow cytometry. (**H**). Western blotting to assess the level of EP procyclin in control and KIN-E RNAi cells. EP procyclin was detected with anti-EP procyclin mAb and FITC-conjugated anti-mouse IgG, whereas TbPSA6 was detected with anti-TbPSA6 pAb and IRDye 680LT anti-rabbit IgG. (**I**). Quantitation of the level of EP procyclin detected by western blotting in panel H. Error bars indicate S.D. calculated from three independent western blotting experiments. **, *p*<0.01; ***, *p*<0.001.

We next investigated the function of KIN-E in the procyclic form of *T*. *brucei* by RNAi. Induction of RNAi by tetracycline causes a gradual decrease of KIN-E protein, which was endogenously tagged with a triple HA epitope, to the level of <10% of the control level after RNAi induction for 4 days and beyond (Fig 3A). Knockdown of KIN-E causes a severe growth defect (Fig 3B) and a drastic change in cell morphology, resulting in the production of epimastigote-like cells among almost 80% of the 1N1K cells after RNAi induction for 48 h (Fig 3C). Epimastigote-like morphology is characterized by a re-positioned kinetoplast, which is either juxtaposed or anterior to the nucleus, and a long unattached flagellum (Fig 3D). Scanning electron microscopy further confirmed the epimastigote-like morphology of the cells with a single flagellum (Fig 3E, panel b). Among the cells with two flagella, ~80% of them possess a long, unattached new flagellum and a normal-length old flagellum (Fig 3E, panle d), and the rest of them possess a long, unattached new flagellum and a long, unattached old flagellum (Fig 3E, panel f). The latter type of two-flagella cells are likely developed from the epimastigote-like one-flagellum cells following further cell cycle progression. While KIN-E RNAi induction for shorter times such as 24 and 48 hours causes major changes in cell morphology, RNAi induction for longer times results in the accumulation of multi-nucleated (>2) cells up to 75% of the total population after 7 days (Fig 3F), suggesting that cytokinesis is arrested after prolonged RNAi induction.

The generation of cells with epimastigote-like morphology by KIN-E RNAi raised the question of whether these epimastigote-like cells, other than morphology resemblance, also possess certain biological features of the epimastigote form. During the development of *T*. *brucei* from the trypomastigote form to the epimastigote form within the gut of tsetse flies, a prominent change is the alteration of the expression of EP and GPEET procyclins [18,19]. During early stages (day 3) of fly infection, only GPEET procyclin is expressed, but later in the infection (day 7), GPEET procyclin disappears and EP procyclin is expressed [19]. EP procyclin is also found in the epimastigote form of *T*. *brucei* [20]. To investigate the relative expression of EP procyclin after KIN-E RNAi, we carried out flow cytometry, which showed that the level of EP procyclin was gradually increased after KIN-E RNAi induction (Fig 3G). Quantitative western blotting showed that the level of EP procyclin was increased to ~3.5-fold of the control level after 3 days of RNAi (Fig 3H and 3I). These results suggest that KIN-E RNAi not only produced cells of epimastigote-like morphology but also altered certain biological features of the trypomastigote cells, such as the expression of EP procyclin, towards the epimastigote form. Since the anti-GPEET procyclin antibody obtained from Cedarlane Labs failed to detect GPEET procyclin, the expression of GPEET procyclin in KIN-E RNAi cells was unable to be investigated.

### KIN-E is required for elongation of the new FAZ

**Fig 4:**
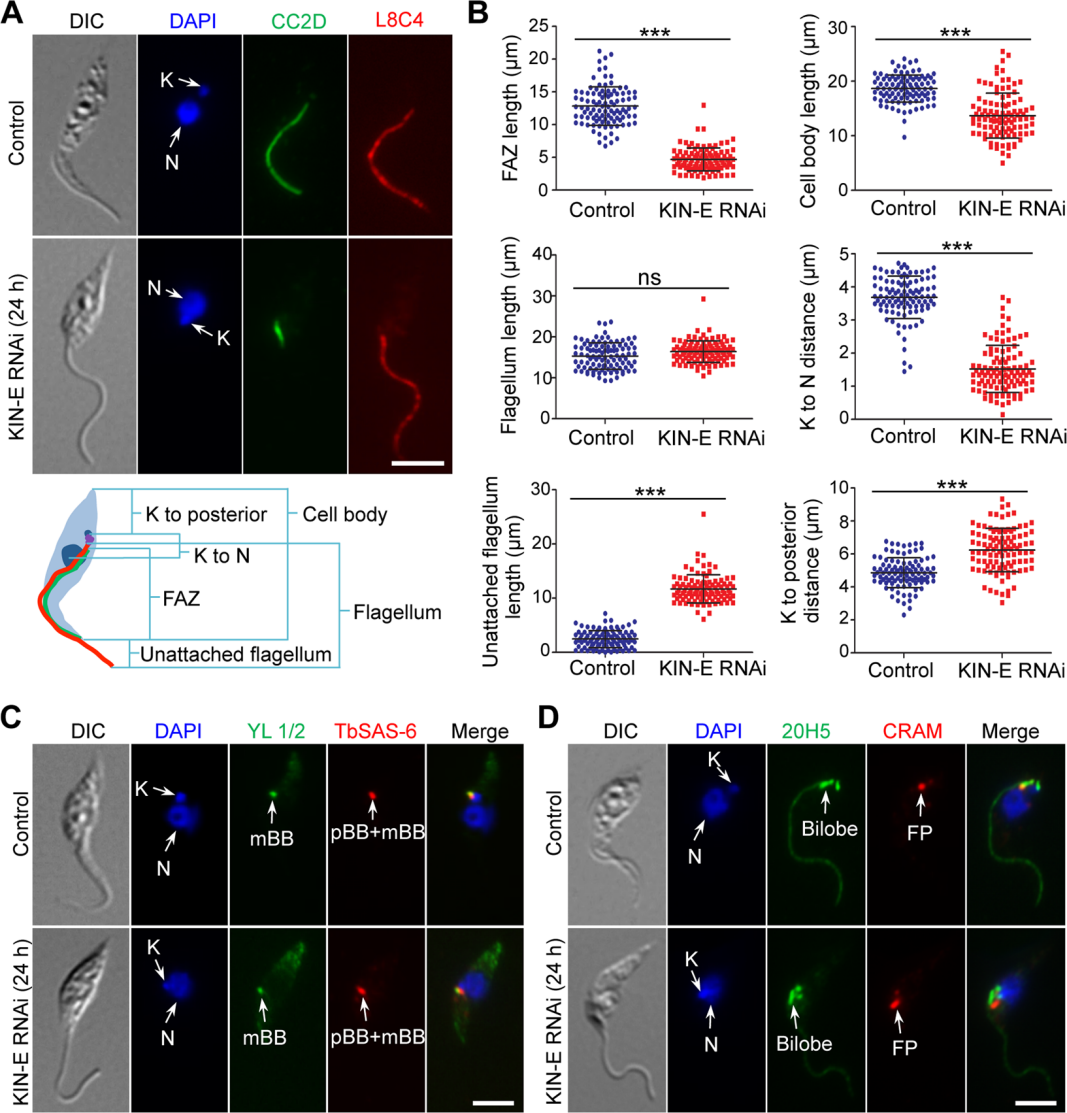
Morphological analysis of the epimastigote-like cells generated by KIN-E RNAi. (**A**). Morphology of 1N1K cells from control and KIN-E RNAi cells. 1N1K cells from the control and KIN-E RNAi-induced population were co-immunostained with anti-CC2D antibody to label the FAZ filament and with anti-PFR2 (L8C4) antibody to label the flagellum. Cells were counterstained with DAPI for nuclear (N) and kinetoplast (K) DNA. The cartoon depicted a 1N1K cell used for measuring the length of cell body, flagellum, unattached flagellum, FAZ filament, kinetoplast (K) to posterior cell tip, and kinetoplast to nucleus (N). Scale bar: 5 μm. (**B**). Morphometric measurement of uninduced control cells and KIN-E RNAi-induced (24 h) cells. 1N1K cells from control and KIN-E RNAi cells (+Tet, 24 h) were immunostained with anti-CC2D and anti-PFR2 antibodies, and the length of the cell body, the FAZ, the flagellum, the unattached flagellum, kinetoplast to nucleus, and kinetoplast to the posterior tip were measured and plotted (*n* = 101 for control cells and *n* = 104 for RNAi cells). ***, *p*<0.001; ns, no statistical significance. (**C**). Position of the flagellar basal body in control and KIN-E RNAi cells. Cells (1N1K) were co-immunostained with YL 1/2 antibody to label the mature basal body (mBB) and with anti-TbSAS-6 antibody to label both the mBB and the pro-basal body (pBB), and then counterstained with DAPI for nuclear and kinetoplast DNA. Scale bar: 5 μm. (**D**). Positions of the bilobed structure and flagellar pocket in control and KIN-E RNAi cells. Cells (1N1K) were co-immunostained with 20H5 antibody to label the bilobed structure and with anti-CRAM antibody to label the flagellar pocket (FP), and then counterstained with DAPI for nuclear and kinetoplast DNA. Scale bar: 5 μm.

Given that the epimastigote-like 1N1K cells from KIN-E RNAi possess a re-positioned kinetoplast and a long, unattached flagellum, we examined whether KIN-E RNAi affected biogenesis and/or elongation of the FAZ and the flagellum. To this end, we immunostained the 1N1K cells with anti-CC2D antibody to label the FAZ and with L8C4 (anti-PFR2 antibody) to stain the paraflagellar rod in the flagellum (Fig 4A), and then measured the length of the FAZ and the flagellum (Fig 4B). Additionally, we also measured the length of the unattached flagellum and the cell body, the distance between kinetoplast and nucleus, and the distance between kinetoplast and cell posterior (Fig 4B). The results showed that the 1N1K cells from KIN-E RNAi possess a significantly shorter FAZ than the control 1N1K (an average length of 4.7 ± 0.3 μm vs 12.9 ± 0.2 μm, *n* = 104 for RNAi cells and *n* = 101 for control cells) (Fig 4A and 4B), and the unattached flagellum of the RNAi cells is more than four times longer than the unattached flagellum of the control cells (an average length of 11.9 ± 1.6 μm vs 2.5 ± 0.2 μm) (Fig 4A and 4B). The 1N1K cells from KIN-E RNAi also appear to be significantly smaller in size than the control 1N1K cells (an average cell body length of 13.7 ± 0.5 μm vs 18.6 ± 0.8 μm) (Fig 4A and 4B). Additionally, the average distance between the kinetoplast and the nucleus is significantly reduced from 3.7 ± 0.4 μm to 1.5 ± 0.1 μm upon KIN-E RNAi (Fig 4B) and consequently, the average distance from the kinetoplast to the cell posterior is increased from 4.9 ± 0.4 μm to 6.2 ± 0.2 μm (Fig 4B). These epimastigote-like 1N1K cells also contain re-positioned cytoskeletal structures, such as the flagellar basal body (Fig 4C), the bilobed structure, and the flagellar pocket (Fig 4D), all of which are anterior to the nucleus rather than posterior to the nucleus as in control cells (Fig 4C and 4D).

**Fig 5:**
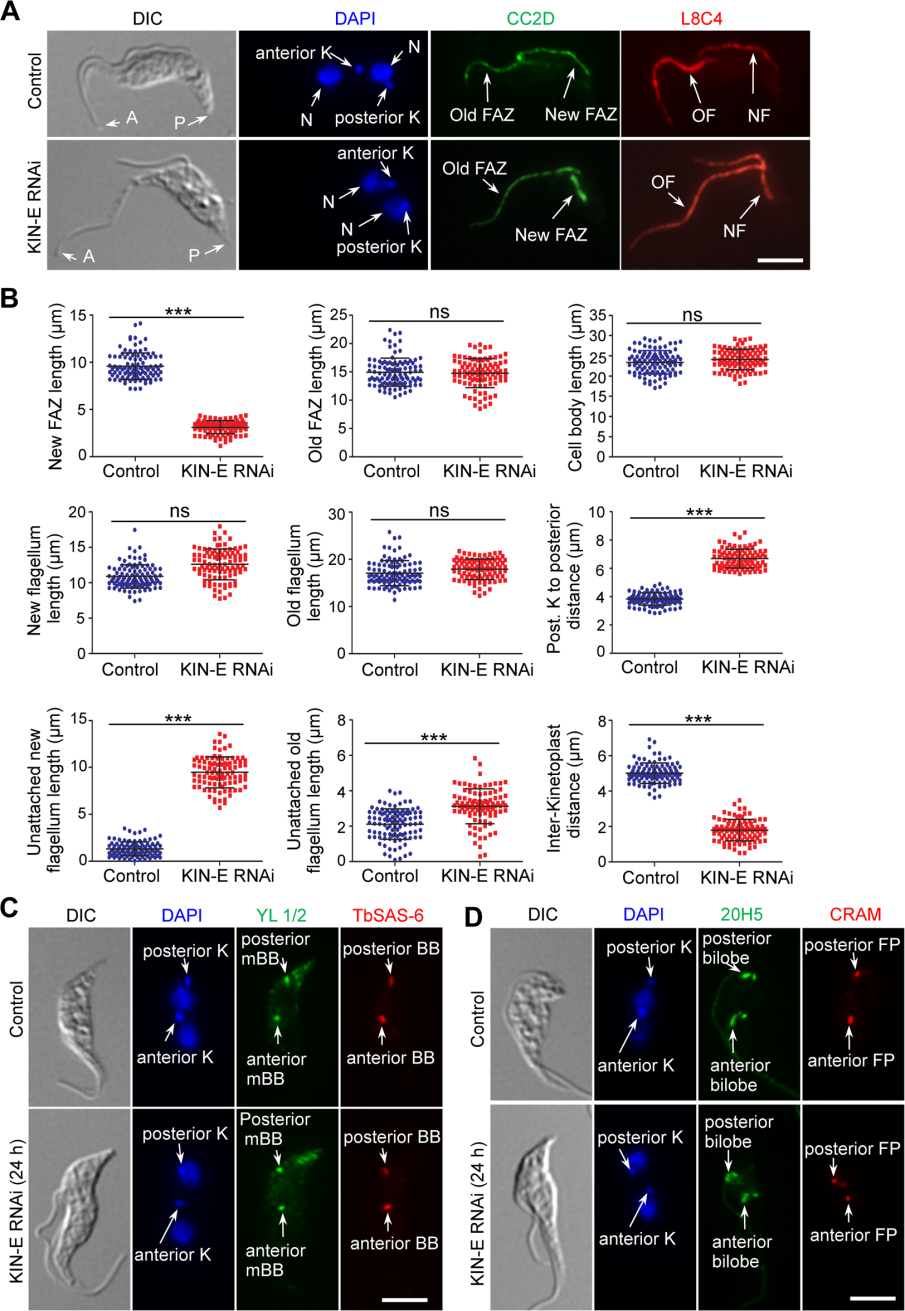
KIN-E RNAi disrupted the elongation of the new FAZ and the migration of the kinetoplast/basal body towards cell posterior. (**A**). Morphology of 2N2K cells from control and KIN-E RNAi cells. 2N2K cells from control and KIN-E RNAi-induced population were immunostained with anti-CC2D and anti-PFR2 (L8C4) antibodies to label the FAZ and the flagellum, respectively. Cells were counterstained with DAPI to stain nuclear (N) and kinetoplast (K) DNA. NF, new flagellum; OF, old flagellum. Scale bar: 5 μm. (**B**). Morphometric measurement of uninduced control cells and KIN-E RNAi-induced (24 h) cells. 2N2K cells from control and KIN-E RNAi cells (+Tet, 24 h) were immunostained with anti-CC2D and anti-PFR2 antibodies. The length of the cell body, the new FAZ, the old FAZ, the new and old flagella, the unattached new and old flagella, posterior kinetoplast to cell posterior distance, and inter-kinetoplast distance were measured and plotted (*n* = 103 for control cells and *n* = 97 for RNAi cells). ***, *p*<0.001; ns, no statistical significance. (**C**). Position of the flagellar basal body in control and KIN-E RNAi cells. Shown are 2N2K cells that were co-immunostained with YL 1/2 antibody to label the mature basal body (mBB) and with anti-TbSAS-6 antibody to lable the total basal body (BB) that is composed of mBB and pro-basal body (pBB). Cells were then counterstained with DAPI for nuclear and kinetoplast DNA. Scale bar: 5 μm. (**D**). Positions of the bilobe structure and the flagellar pocket in control and KIN-E RNAi cells. Shown are 2N2K cells that were co-immunostained with 20H5 antibody and anti-CRAM antibody to label the bilobe structure and the flagellar pocket (FP), respectively, and then counterstained with DAPI for nuclear and kinetoplast DNA. Scale bar: 5 μm.

The morphological changes observed in 1N1K cells are also detected in 2N2K cells (Fig 5). The new FAZ of the RNAi cells is significantly shorter than that of the control cells (an average length of 3.1 ± 0.08 μm vs 9.6 ± 0.7 μm, *n* = 97 for RNAi cells and *n* = 103 for control cells), and the new, unattached flagellum of the RNAi cells is significantly longer than that of the control cells (an average length of 9.5 ± 0.2 μm vs 1.32 ± 0.07 μm) (Fig 5A and 5B). However, the old FAZ and the old flagellum are not affected (Fig 5A and 5B), indicating that RNAi of KIN-E only disrupts the elongation of the new FAZ. It should be noted that some 2N2K cells also possess a longer unattached old flagellum (Figs 5B and 3E), but these cells likely are developed from the 1N1K cells with a longer unattached flagellum. Another change observed in the 2N2K cells is the reduced inter-kinetoplast distance (an average length of 1.8 ± 0.1 μm in RNAi cells vs 5.02 ± 0.01 μm in control cells) and the increased distance between the posterior kinetoplast to the cell posterior (an average length of 6.7 ± 0.2 μm in RNAi cells vs 3.8 ± 0.1 μm in control cells) (Fig 5A and 5B), suggesting that migration of the posterior kinetoplast is defective. Moreover, there is also a defective migration of other cytoskeletal structures, such as the posterior basal body (Fig 5C), the posterior bilobe structure (Fig 5D), and the posterior flagellar pocket (Fig 5D). These results demonstrated that RNAi of KIN-E abolishes the migration of the posterior kinetoplast and its associated basal body, the posterior bilobe structure, and the posterior flagellar pocket towards the cell posterior. This likely is due to the defective elongation of the new FAZ (Fig 5A), as it was previously proposed that basal body positioning is controlled by FAZ elongation [21].

### The structural motifs in KIN-E required for KIN-E function

**Fig 6:**
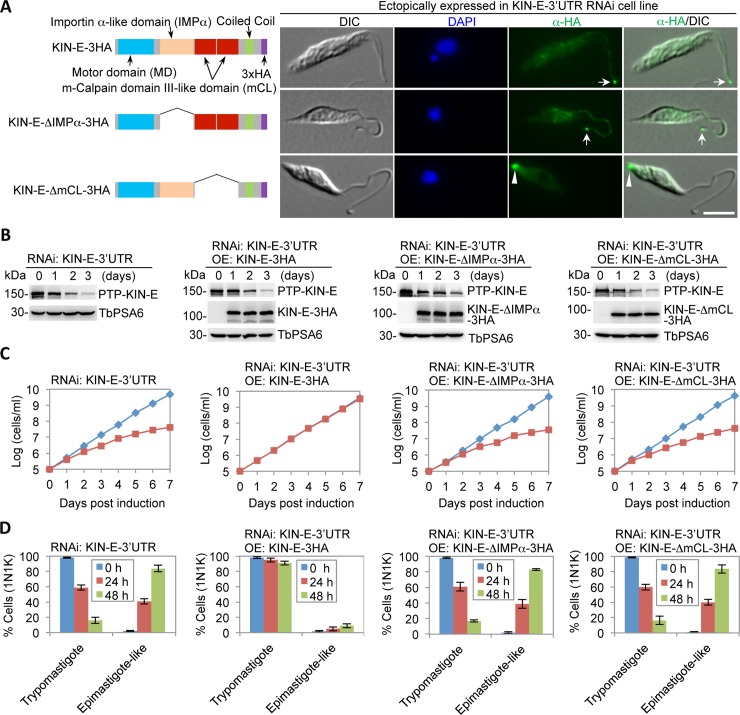
The structural motifs required for KIN-E function. (**A**). Subcellular localization of ectopically expressed KIN-E and its various mutants in KIN-E 3’UTR RNAi cell line. Wild-type KIN-E, the importin α-like domain deletion mutant (KIN-E-ΔIMPα), and the m-calpain domain III (mCL#1& mCL#2) deletion mutant (KIN-E-ΔmCL) were each tagged with a triple HA epitope at the C-terminus and ectopically expressed in KIN-E-3’UTR RNAi cell line. Arrows indicate KIN-E and KIN-E-ΔIMPα at the flagellar tip, whereas the arrowhead indicates KIN-E-ΔmCL at the posterior tip. Scale bar: 5 μm. (**B**). Western blotting to monitor the knockdown of endogenous KIN-E, which was tagged with an N-terminal PTP epitope, and ectopically expressed wild-type and mutant KIN-E proteins, which were tagged with a C-terminal triple HA epitope. TbPSA6 served as the loading control. (**C**). Complementation of KIN-E-3’UTR RNAi by 3HA-tagged KIN-E and its mutants. Shown are the growth curves of KIN-E-3’UTR RNAi cell line and the KIN-E-3’UTR RNAi cell lines expressing wild-type or mutant KIN-E proteins at an ectopic locus. (**D**). Quantification of 1N1K cells of trypomastigote morphology and epimastigote-like morphology in KIN-E-3’UTR RNAi cell line and RNAi complementation cell lines. A total of 100 1N1K cells were counted for each time point, and three repeats were performed. Error bars indicate S.D.

The requirement of the two unusual structural motifs for KIN-E function was investigated using the KIN-E RNAi complementation cell lines in which wild-type KIN-E or KIN-E mutants were ectopically expressed in KIN-E-3’UTR RNAi background. RNAi of KIN-E by targeting its 3’UTR and ectopic expression of KIN-E or its mutants were induced by incubating with tetracycline. Ectopically expressed KIN-E and KIN-E-ΔIMPα in KIN-E-3’UTR RNAi cells localized to the flagellar tip, whereas KIN-E-ΔmCL localized to the posterior tip of the cell (Fig 6A). Knockdown of endogenous KIN-E protein, which was tagged with an N-terminal PTP epitope, and ectopic overexpression of KIN-E and its mutants, which were tagged with a C-terminal triple HA epitope, were confirmed by western blotting (Fig 6B). RNAi of KIN-E by targeting the 3’UTR of KIN-E also causes a growth defect (Fig 6C) and produces epimastigote-like cells (Fig 6D), similar to the RNAi by targeting the coding region of KIN-E (Fig 3B and 3C). Ectopic expression of wild-type KIN-E in KIN-E-3’UTR RNAi background restores cell growth to the rate of non-induced control cells (Fig 6C) and restores trypomastigote morphology (Fig 6D), demonstrating the rescue of the RNAi cells by ectopically expressed KIN-E. Expression of KIN-E-ΔIMPα and KIN-E-ΔmCL, however, does not restore cell growth (Fig 6C) and still produces epimastigote-like cells (Fig 6D), indicating that the importin α-like domain and the m-calpain domain III-like domains are required for KIN-E function.

### KIN-E interacts with FLAM3 and is required for targeting FLAM3 to the flagellum

**Fig 7:**
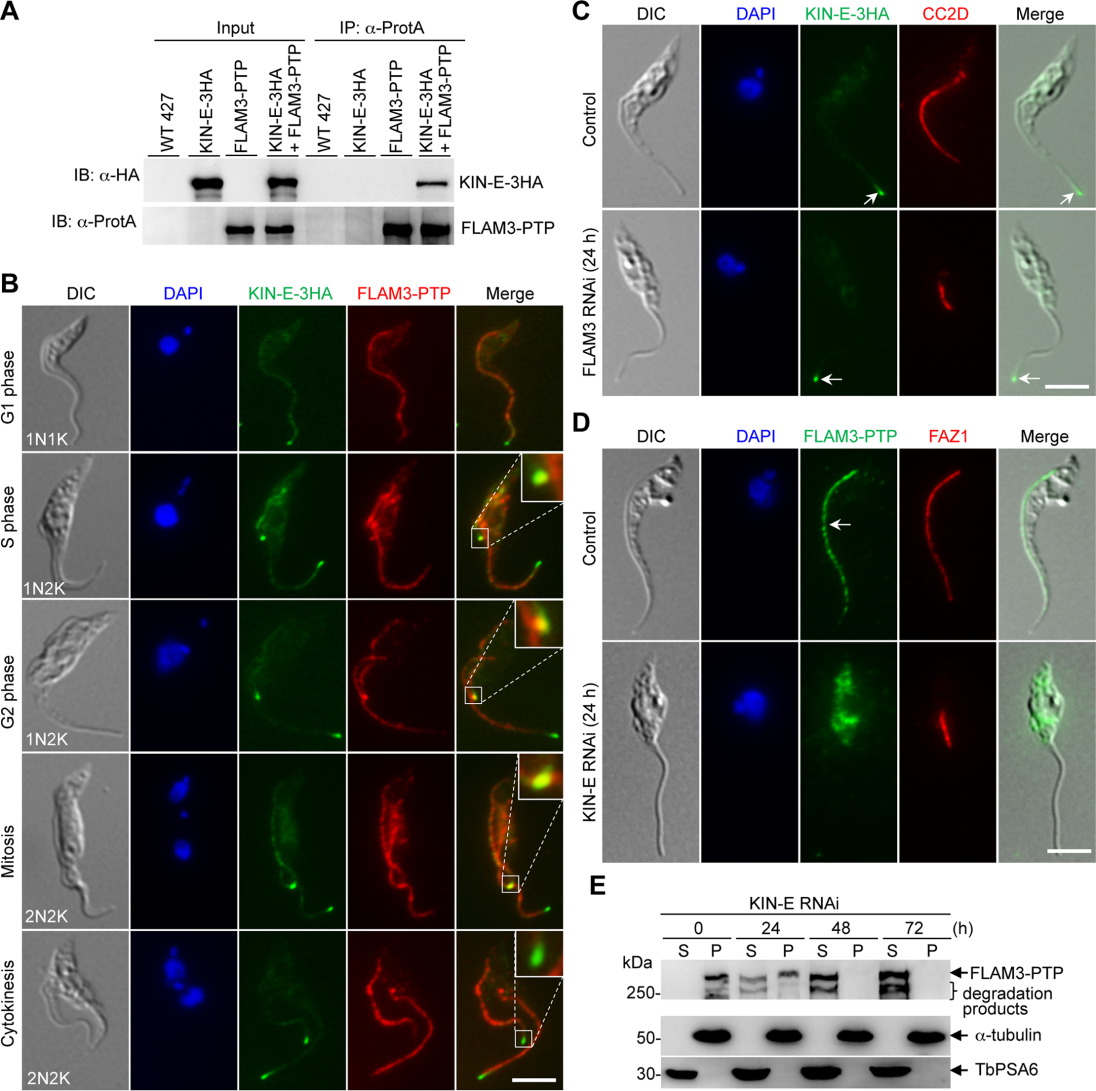
KIN-E interacts with FLAM3 and is required for FLAM3 localization to the flagellum. (**A**). Immunoprecipitation of FLAM3-PTP pulled down KIN-E-3HA from trypanosome cell lysate. Wild-type 427 cells, cells expressing endogenously 3HA-tagged KIN-E, and cells co-expressing endogenously PTP-tagged FLAM3 and 3HA-tagged KIN-E were lysed, and cleared cell lysate was incubated with IgG beads to pull down FLAM3-PTP and its associated proteins. Immunoprecipitates were immunoblotted by anti-HA antibody and anti-Protein A (α-ProtA) antibody to detect TbKIN-E-3HA and FLAM3-PTP, respectively. (**B**). KIN-E co-localizes with FLAM3 at the flagella connector region. Cells co-expressing endogenously 3HA-tagged KIN-E and PTP-tagged FLAM3 were co-immunostained with FITC-conjugated anti-HA mAb and anti- Protein A pAb, and counterstained with DAPI for nuclear and kinetoplast DNA. Scale bar: 5 μm. (**C**). Effect of FLAM3 depletion on KIN-E localization. KIN-E was endogenously tagged with a tripe HA epitope in FLAM3 RNAi cell line. Control and RNAi-induced cells were immunostained with FITC-conjugated anti-HA antibody to detect KIN-E-3HA and with anti-CC2D antibody to label the FAZ. Arrows indicated the enrichment of KIN-E at the flagellar tip. Scale bar: 5 μm. (**D**). Effect of KIN-E RNAi on FLAM3 localization. FLAM3 was endogenously tagged with a PTP epitope in KIN-E RNAi cell line. Control and RNAi-induced cells were immunostained with anti-Protein A (α-ProtA) antibody to detect FLAM3-PTP and with anti-FAZ1 (L3B2) antibody to label the FAZ. Arrows indicated the FLAM3-PTP signal in the flagellum. Scale bar: 5 μm. (**E**). Effect of KIN-E RNAi on the distribution of FLAM3 in cytosolic and cytoskeletal fractions. Control and KIN-E RNAi cells expressing endogenously PTP-tagged FLAM3 were lysed in PEME buffer containing 1% NP-40. Cell lysate was spun down to separate cytosolic soluble (S) fraction and cytoskeletal pellet (P) fraction for western blotting with anti-Protein A (α-ProtA) antibody to detect FLAM3-PTP in the two fractions. The same membrane was probed with anti-α-tubulin antibody and anti-PSA6 antibody to serve as cytoskeleton and cytosol markers, respectively.

Previous studies showed that depletion of two flagellum FAZ domain proteins, ClpGM6 and FLAM3, causes major changes in cell morphology in the trypomastigote form of *T*. *brucei* [4,5], similar to the phenotypes caused by KIN-E RNAi (Figs 3–5), suggesting that KIN-E may function in the same pathway as ClpGM6 and FLAM3. To test whether KIN-E interacts with the two proteins, we carried out co-immunprecipitation experiments. Our numerous attempts to tag ClpGM6 with a triple HA epitope at either the N-terminus or the C-terminus failed due to unknown reasons; therefore, our efforts were focused on FLAM3. Immunoprecipitation of FLAM3, which was tagged with a C-terminal PTP epitope from its endogenous locus, is able to pull down KIN-E, which was endogenously tagged with a triple HA epitope in the same cell line (Fig 7A), indicating that KIN-E interacts with FLAM3 *in vivo* in *T*. *brucei*.

Given that KIN-E interacts with FLAM3 *in vivo* in *T*. *brucei*, we investigated their co-localization. FLAM3 localizes to the flagellum and the flagella connector, but its level is much reduced at the unattached flagella near the distal flagellar tips [4,14]. At the distal tip of the old flagellum, KIN-E does not co-localize with endogenously PTP-tagged FLAM3 (Fig 7B), in agreement with the lack of FLAM3 at the old flagellar tip in 1N2K and 2N2K cells [14]. At the flagella connector in 1N2K and 2N2K cells during late S phase to mitosis, however, KIN-E co-localizes with FLAM3 (Fig 7B). During cytokinesis when the flagella connector is dissolved, KIN-E at the tip of the new flagellum does not co-localize with FLAM3 (Fig 7B). It should be noted that KIN-E in the flagella, albeit at a lower level, also co-localize with FLAM3 except at the unattached flagella near the distal tips (Fig 7B).

We next asked whether FLAM3 is required for KIN-E localization. To this end, KIN-E was endogenously tagged with a C-terminal triple HA epitope in cells harboring the FLAM3 RNAi construct. Control and FLAM3 RNAi cells were then immunostained with anti-HA antibody to detect TbKIN-E-3HA and anti-CC2D antibody to label the FAZ. Efficient knockdown of FLAM3 was confirmed by western blotting (S1 Fig), and depletion of FLAM3 produces cells with epimastigote-like morphology (Fig 7C), similar to the previous report [4]. In these epimastigote-like cells, KIN-E is still detectable in the flagellum and enriched at the distal tip of the flagellum in all (>1,000) of the cells examined (Fig 7C, arrow), demonstrating that FLAM3 is not required for KIN-E localization.

Conversely, the effect of KIN-E depletion on FLAM3 localization was also examined. FLAM3 was endogenously tagged with a PTP epitope in cells harboring the KIN-E RNAi construct, and immunofluorescence microscopic analysis of control and KIN-E RNAi-induced cells showed that KIN-E RNAi disrupts the localization of FLAM3, causing its mis-localization to the cytosol (Fig 7D). The effect on the subcellular distribution of FLAM3 was further investigated by western blotting, which showed that FLAM3 is partly distributed to the soluble cytosolic fraction upon KIN-E RNAi for 24 h and then exclusively distributed to the cytosol after RNAi induction for 48 h and beyond (Fig 7E), in contrast to its exclusive distribution in the cytoskeletal pellet fraction in non-induced control cells (Fig 7E). Together, these results suggest that KIN-E is required for targeting FLAM3 to the flagellum.

### The structural motif in KIN-E required for interacting with and targeting FLAM3

**Fig 8:**
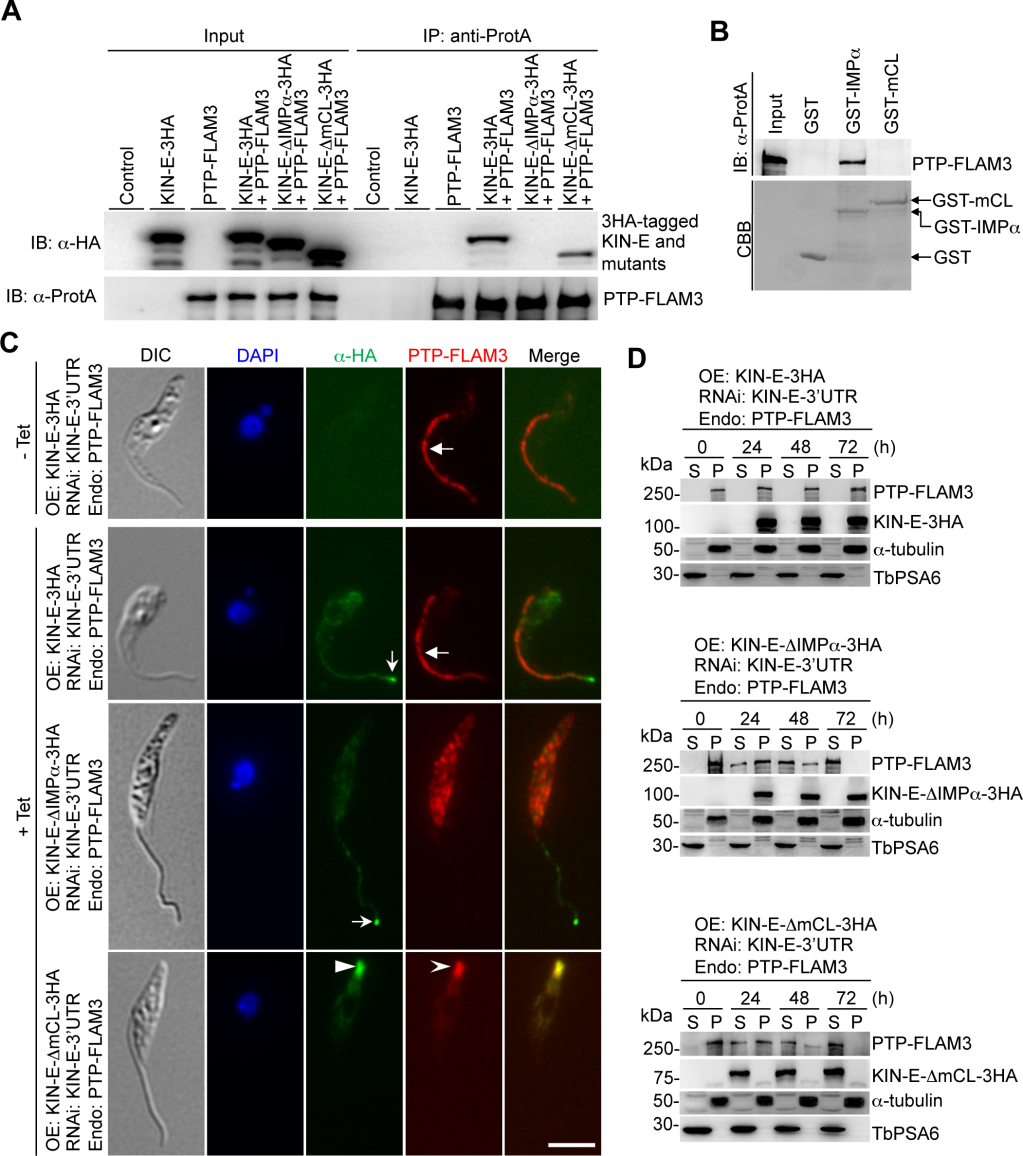
The structural motifs in KIN-E required for FLAM3 interaction and localization. (**A**). Co-immunoprecipitation to examine the requirement of KIN-E structural motifs for interaction with FLAM3. FLAM3 was endogenously tagged with an N-terminal PTP epitope in KIN-E-3’UTR RNAi cells expressing 3HA-tagged wild-type and mutant KIN-E proteins. PTP-FLAM3 was precipitated by IgG beads, and the immunoprecipitated proteins were immunoblotted with anti-HA antibody to detect 3HA-tagged KIN-E and its mutants and with anti-Protein A (α-ProtA) antibody to detect PTP-FLAM3. Cells expressing PTP-FLAM3 only and KIN-E-3HA only were included as controls. (**B**). *In vitro* interaction of the importin-α-like domain of KIN-E with FLAM3. Shown are the GST pull-down results using purified recombinant GST-fused importin-α-like domain (IMPα) and m-calpain domain III-like domain (mCL) as the baits. GST alone served as the negative control. FLAM3 was tagged with an N-terminal PTP epitope and detected by anti-Protein A (α-ProtA) antibody, whereas GST and GST fusion proteins were detected by Coomassie Brilliant Blue (CBB) staining. (**C**). The requirement of KIN-E structural motifs for FLAM3 localization. The same cell lines used in panel A were used for immunofluorescence microscopy. Non-induced (-Tet) and tetracycline-induced (+Tet, 24 h) cells were immunostained with anti-Protein A (α-ProtA) pAb to detect PTP-FLAM3 and FITC-conjugated anti-HA mAb to detect 3HA-tagged KIN-E, KIN-E-ΔIMPα, or KIN-E-ΔmCL. Scale bar: 5 μm. (**D**). Subcellular distribution of FLAM3 protein in KIN-E-3’UTR RNAi cells expressing wild-type and mutant KIN-E proteins. Un-induced and tetracycline-induced cells were lysed in PEME buffer containing 1% NP-40. Cell lysate was spun down to separate cytosolic soluble (S) fraction and cytoskeletal pellet (P) fraction for western blotting with anti-Protein A antibody to detect PTP-FLAM3 and with anti-HA antibody to detect 3HA-tagged KIN-E and its mutants in the two fractions. The same membrane was re-probed with anti-α-tubulin antibody and anti-PSA6 antibody to serve as cytoskeleton and cytosol markers, respectively.

The generation of KIN-E RNAi complementation cell lines allowed us to investigate which domain(s) in KIN-E is required for interaction with FLAM3. To this end, FLAM3 was endogenously tagged with an N-terminal PTP epitope in the RNAi complementation cell lines described above, and co-immunoprecipitation was carried out. The results showed that immunoprecipitation of FLAM3 pulls down wild-type KIN-E and KIN-E-ΔmCL, but not KIN-E-ΔIMPα (Fig 8A), indicating that the importin-α-like domain is required for interacting with FLAM3. We next carried out GST pull-down experiments using purified recombinant GST-fused importin-α-like domain and GST-fused m-calpain domain III-like domain as the baits, and the results showed that the importin-α-like domain, but not the m-calpain domain III-like domain pulls down FLAM3 (Fig 8B), demonstrating that the importin-α-like domain mediates the interaction between KIN-E and FLAM3.

We next examined the localization of FLAM3 in KIN-E mutants by immunofluorescence microscopy. In KIN-E-3’UTR RNAi cells expressing 3HA-tagged KIN-E, PTP-tagged FLAM3 localizes to the flagellum, as in the non-induced control cells (Fig 8C) and as reported previously [4,14]. However, in KIN-E-3’UTR RNAi cells expressing 3HA-tagged KIN-E-ΔIMPα, PTP-tagged FLAM3 is mis-localized to the cytosol (Fig 8C) despite that KIN-E-ΔIMPα still localizes to the flagellum and the flagellar tip (Fig 8C). This result is consistent with the observation that KIN-E-ΔIMPα does not interact with FLAM3 (Fig 8A). In KIN-E-3’UTR RNAi cells expressing KIN-E-ΔmCL, FLAM3 is detected at the posterior end of the cell, where it co-localizes with KIN-E-ΔmCL (Fig 8C). The re-distribution of FLAM3 from the flagellum to the posterior cell end likely is attributed to the re-distribution of KIN-E-ΔmCL, which is still capable of interacting with FLAM3 (Fig 8A).

The distribution of FLAM3 between cytosolic and cytoskeletal fractions in these RNAi complementation cell lines was assessed by western blotting. In non-induced control cells and KIN-E-3’UTR RNAi cells expressing wild-type KIN-E, FLAM3 is detected in the insoluble, cytoskeletal fraction (Fig 8D). However, in KIN-E-3’UTR RNAi cells expressing KIN-E-ΔIMPα, FLAM3 is gradually shifted to the soluble, cytosolic fraction upon tetracycline induction and is exclusively in the soluble fraction after induction for 72 h (Fig 8D), consistent with its subcellular localization to the cytosol (Fig 8C). In these cells, KIN-E-ΔIMPα is still detected in the cytoskeletal fraction (Fig 8D), similar to the wild-type KIN-E (Fig 8D), suggesting the dissociation of FLAM3 from KIN-E-ΔIMPα due to lack of interactions (Fig 8A). In KIN-E-3’UTR RNAi cells expressing KIN-E-ΔmCL, FLAM3 is detected in the soluble, cytosolic fraction, together with KIN-E-ΔmCL (Fig 8D), suggesting that at the posterior cell tip, the mis-localized FLAM3 and KIN-E-ΔmCL either do not associate with the microtubule cytoskeleton or associate with the cytoskeleton in a manner that cannot tolerate detergent treatment.

## Discussion

Morphology transitions during trypanosome life cycle development appear to involve the modulation of the length of the FAZ through a cohort of FAZ flagellum domain proteins, such as ClpGM6 [5] and FLAM3 [4,14], and some intracellular FAZ proteins, such as FAZ9 [6] and TbSAS-4 [7]. Here an orphan kinesin, KIN-E, was found to play an essential role in controlling morphology transitions in *T*. *brucei* (Fig 3). KIN-E is unusual in that it contains in its C-terminus an importin α-like domain and two m-calpain domain III-like domains (Fig 1), which have not been found in any other kinesins in any organisms. The importin α-like domain and the m-calpain domain III-like domains play distinct roles in regulating KIN-E function. The importin α-like domain is not required for KIN-E localization (Fig 6A), but it is essential for KIN-E function (Fig 6C) and for interacting with FLAM3 and transporting the latter to the flagellum (Fig 8C). The m-calpain domain III-like domains are not involved in binding to FLAM3 (Fig 8A and 8B), but are essential for KIN-E localization to the flagellum (Fig 8C) and for KIN-E function (Fig 6C). The domain III in calpain, a calcium-dependent cysteine protease in vertebrates, functions as a calcium-regulated phospholipid-binding domain [22]. Thus, the two m-calpain domain III-like domains in KIN-E may also be capable of binding to calcium and lipid. The biochemical function and the mechanistic role of the m-calpain domain III-like domains in mediating KIN-E localization require further investigation.

Most kinesins are microtubule plus end-directed motor proteins that transport cargos along the microtubules to their cellular destinations [8]. The enrichment of KIN-E at the distal tip of the flagellum (Fig 2), where the plus ends of the axonemal microtubules are located, and the localization of the KIN-E-ΔmCL mutant to the posterior tip of the cell (Fig 6A), where the plus ends of the subpellicular microtubules are located [23], suggest that KIN-E is plus end-directed and may transport cargos toward the distal tip of the flagellum along the axonemal microtubules. FLAM3 has been identified as one of the cargos that KIN-E transports to the flagellum. This important point was demonstrated by several lines of evidence. First, FLAM3 interacts with KIN-E *in vivo* and co-localizes with KIN-E at the flagella connector region and, to a less extent, along the most part of the flagellum (Fig 7A and 7B). Second, depletion of KIN-E disrupts FLAM3 localization, resulting in the distribution of FLAM3 to the cytosol (Fig 7D and 7E). Finally, in cells expressing the KIN-E-ΔmCL mutant that still interacts with FLAM3 (Fig 8A), FLAM3, together with KIN-E-ΔmCL, is re-directed to the posterior end of the cell (Fig 8C). In contrast, in cells expressing the KIN-E-ΔIMPα mutant that still localizes to the flagellar tip but does not interact with FLAM3, FLAM3 is not targeted to the flagellum (Fig 8C), providing another line of evidence to demonstrate that targeting of FLAM3 to the flagellum depends on interaction with KIN-E.

Proteins and vesicles that are transported into the flagellum need to pass through the transition zone, a structural junction between the basal body and the flagellar/ciliary axoneme [24]. At the proximal end of the transition zone, a terminal plate crosses the transition zone, and it contains pores for the passage of intraflagellar transport (IFT) trains, which deliver axonemal components to the flagellar tip [24]. At the distal end of the basal body, striated transitional fibers radiate to join the plasma membrane [25], and the junction between the transitional fibers and the plasma membrane serves as a docking site for IFT trains [26]. We speculate that KIN-E may carry FLAM3 and other unidentified cargos through the transition zone [27] to deliver cargos to the flagellum. Given the requirement of the m-calpain domain III-like domains for KIN-E localization (Figs 2C and 6A), we postulate that these domains may mediate the trafficking of KIN-E and its cargos to the transition zone or the loading of them onto the transitional fibers before passing through the terminal plate of the transition zone to enter the flagellum. Within the flagellum/cilium, the axonemal microtubule doublets function as double-track railways to transport cargos bi-directionally, with the B-microtubules transporting anterograde IFT trains and the A-microtubules transporting retrograde IFT trains [28]. In trypanosome flagellum, anterograde IFT trains also move on a restricted set of axonemal microtubules [29]. It is thus possible that KIN-E also travels along the B-microtubules of the axonemal microtubule doublets to deliver FLAM3 and other cargos to the distal tip of the flagellum for elongation of the FAZ.

KIN-E-mediated transport of FLAM3 likely is independent of the Kinesin-2-mediated IFT system in *T*. *brucei* flagellum [30], as IFT is essential for flagellum assembly [31], but KIN-E is not required for flagellum formation (Fig 5A and 5B). KIN-E also appears to act independently of the flagella connector [32] that functions at the membrane junction between the new and old flagella, despite it partly overlaps with the flagella connector 1 (FC1) protein (Fig 2B), as KIN-E is not identified by flagella connector protein 1 (FCP1) immunoprecipitation [33] and depletion of KIN-E does not disrupt the connection of the new flagellum to the old flagellum (Fig 5C and 5D).

RNAi of KIN-E, FLAM3, and ClpGM6 all produce epimastigote-like cells, but there appear to be significant differences in cell proliferation between ClpGM6 RNAi cells and KIN-E and FLAM3 RNAi cells. Knockdown of ClpGM6 does not affect cell proliferation [5], whereas depletion of KIN-E or FLAM3 inhibits cell proliferation and generates multi-nucleated cells (Fig 3F and [4,14]). Although the detailed mechanism underlying this distinction is unknown, the phenotypic difference may be attributed to their differential effect on the length of the FAZ. RNAi of ClpGM6 produces epimastigote-like cells that contain a FAZ of 5–10 μm in length [5], whereas RNAi of FLAM3 [4] and RNAi of KIN-E (Fig 4) both produce epimastigote cells that contain a FAZ of less than 3 μm in length. It was proposed that the critical minimum length of the FAZ is 3 μm, and the FAZ of less than 3 μm in length is unable to support cytokinesis [4]. In this regard, KIN-E and FLAM3 appear to function similarly in controlling morphology transitions and cell proliferation. Interestingly, previous work showed that ClpGM6 and FLAM3 are co-immunoprecipitated from trypanosome lysate and are interdependent in maintaining their protein levels [4]. The current work showed that KIN-E and FLAM3 interact in trypanosomes and FLAM3 localization depends on KIN-E, but not vice versa (Fig 7). Given the effect of KIN-E depletion on FLAM3 and the interdependence between FLAM3 and ClpGM6, it is possible that KIN-E RNAi may also affect ClpGM6 localization in an indirect manner through disrupting FLAM3 localization (if ClpGM6 is not a cargo for KIN-E). It would also be interesting to investigate whether ClpGM6 is similarly targeted to the flagellum by KIN-E.

The morphological differences between the trypomastigote form and the epimastigote form lie in the length of the FAZ and the position of the mitochondrial genome, the flagellar pocket, the flagellum, and flagellum-associated structures relative to the nucleus [1]. KIN-E-deficient cells possess characteristic features of the epimastigote form with a shorter FAZ and re-positioned organelles/cytoskeletal structures, including flagellum, flagellar pocket, basal body, bilobe, and kinetoplast, to the anterior of nucleus (Figs 3 and 4). The fact that depletion of KIN-E in trypomastigote cells produces epimastigote-like cells suggests that KIN-E functions to maintain trypomastigote cell morphology, likely by promoting FAZ elongation and positioning organelles and cytoskeletal structures. However, given that FAZ elongation appears to control basal body positioning [21], it is likely that the defects in organelle/cytoskeletal structure positioning caused by KIN-E depletion are attributed to defective FAZ elongation, which is attributed to the failure to target FLAM3, a known FAZ flagellum domain protein required for FAZ elongation and life cycle form morphology transitions [4,14], to the flagellum (Fig 7). The trypomastigote and epimastigote forms also differ in certain biological features, including the distinct expression levels of the procyclins. During the development of *T*. *brucei* from the trypomastigote form to the epimastigote form in the gut of tsetse flies, the level of GPEET procyclin gradually decreases, but the level of EP procyclin gradually increases [19]. Intriguingly, KIN-E RNAi cells also have higher levels of EP procyclin (Fig 3G–3I), suggesting that the epimastigote-like cells produced by depletion of KIN-E also possess certain biological features of the epimastigote form.

In summary, we identified a novel function of the orphan kinesin KIN-E in controlling morphology transitions in *T*. *brucei* and uncovered the mechanistic role of KIN-E in targeting the FAZ flagellum domain protein FLAM3 to the flagellum to promote FAZ elongation, thereby maintaining flagellum-cell body attachment and positioning the flagellum and flagellum-associated cytoskeletal structures to assume trypomastigote cell morphology.

## Materials and methods

### Trypanosome cell culture

The procyclic form of *T*. *brucei* strain 427 was grown at 27°C in SDM-79 medium containing 10% heat-inactivated fetal bovine serum. The procyclic form of *T*. *brucei* strain 29–13 [34] was cultured at 27°C in SDM-79 medium supplemented with 10% heat-inactivated fetal bovine serum (Atlanta Biologicals, Inc), 15 μg/ml G418, and 50 μg/ml hygromycin. Cells were sub-cultured by 1/10 dilution with fresh medium whenever the cell density reached 5×10^6^/ml.

### RNA interference

To generate the KIN-E RNAi cell line, a 623-bp DNA fragment (nucleotides 235–866) corresponding to the N-terminal coding region of KIN-E was cloned into the pZJM vector [35]. To generate the FLAM3 RNAi cell line, a 938-bp DNA fragment (nucleotides 1004–1941) corresponding to the middle portion of the coding region of FLAM3 was cloned into the pZJM vector. The same DNA fragment of *FLAM3* gene was used for RNAi previously [4]. The pZJM-KIN-E and pZJM-FLAM3 plasmids were each linearized with NotI and transfected into the 29–13 strain by electroporation. Transfectants were selected with 2.5 μg/ml phleomycin in SDM-79 medium containing 15 μg/ml G418 and 50 μg/ml hygromycin, and then cloned by limiting dilution in a 96-well plate. RNAi was induced by incubating the cells with 1.0 μg/ml tetracycline. Growth of cells was monitored daily by counting the cells with a hemacytometer. Three independent clones were selected and analyzed, which all generated almost identical phenotypes. Only the results obtained from characterizing one clone was presented.

### KIN-E RNAi by targeting the 3’UTR and complementation of KIN-E RNAi

For RNAi complementation experiments, a new KIN-E RNAi cell line was generated by targeting against the 3’UTR of KIN-E. A 618-bp fragment from the 3’UTR of *KIN-E* gene, which does not overlap with the downstream gene, was cloned into the pZJM-PAC vector. The resulting construct was linearized with NotI, and transfected into the 29–13 cell line. Transfectants were selected under 1 μg/ml puromycin in addition to 15 μg/ml G418 and 50 μg/ml hygromycin B, and further cloned by limiting dilution in a 96-well plate.

Full-length *KIN-E* gene was cloned into pLew100-3HA-BLE vector, which bears the actin 3’-UTR. *KIN-E* gene sequence with the deletion of the importin α-like domain (a.a. 413–684) and *KIN-E* gene sequence with the deletion of the m-calpain domain III-like domains (a.a. 713–997) were each cloned into the pLew100-3HA-BLE vector for expression of 3HA-tagged KIN-E-ΔIMPα and KIN-E-ΔmCL, respectively. These plasmids were each linearized with NotI and transfected into the cell line containing the pZJM-TbKIN-E-3’UTR-PAC construct. Transfectants were selected under 2.5 μg/ml phleomycin in addition to 1 μg/ml puromycin, 15 μg/ml G418, and 50 μg/ml hygromycin B, and cloned by limiting dilution in a 96-well plate. These plasmids were also transfected into the 29–13 cell line for determining the localization of ectopically overexpressed proteins.

### *In situ* epitope tagging of proteins

Epitope tagging of KIN-E, FLAM3 [14], and FC1 [6] from their respective endogenous locus was carried out using the PCR-based method [36]. To examine whether epitope-tagging of KIN-E disrupts its function, the second allele of KIN-E was knocked out by replacing the coding sequence with puromycin-resistance gene. The resulting cell line grows normally like the wild-type 427 cell line. Epitope tagging of FLAM3 and FC1 does not disrupt their function in previous reports [6,14]. For KIN-E and FLAM3 co-localization, KIN-E was tagged with a C-terminal triple HA epitope (neomycin resistance) and FLAM3 was tagged with a C-terminal PTP epitope (puromycin resistance) in the 427 cell line. For KIN-E and FC1 co-localization, KIN-E was tagged with a triple HA epitope (neomycin resistance) and FC1 was tagged with a PTP epitope (puromycin resistance) in the 427 cell line. Transfectants were selected under 40 μg/ml G418 and 1 μg/ml puromycin, and cloned by limiting dilution in a 96-well plate.

KIN-E was also tagged with a C-terminal triple HA epitope (puromycin resistance) in the 29–13 cell line containing the pZJM-KIN-E RNAi construct or the pZJM-FLAM3 RNAi construct. FLAM3 was tagged with a C-terminal PTP epitope (puromycin resistance) in the 29–13 cell line containing the pZJM-KIN-E RNAi construct. Transfectants were selected under 1 μg/ml puromycin in addition to 15 μg/ml G418 and 50 μg/ml hygromycin B, and further cloned by limiting dilution in a 96-well plate.

FLAM3 was also tagged with an N-terminal PTP epitope (blasticidin resistance) in the 29–13 cell line containing the pZJM-KIN-E-3’UTR-PAC RNAi construct, pZJM-FLAM3 RNAi cell line, and the pLew100-3HA-BLE construct for ectopic expression of 3HA-tagged wild-type KIN-E or various KIN-E mutants. Transfectants were selected under 10 μg/ml blasticidin in addition to 15 μg/ml G418, 50 μg/ml hygromycin B, 2.5 μg/ml phleomycin, and 1 μg/ml puromycin, and cloned by limiting dilution in a 96-well plate.

### Flow cytometry and quantitative western blotting

Flow cytometry analysis of the expression of EP procyclin in control and KIN-E RNAi cells was carried out according to the procedure described previously [37]. EP procyclin was detected using the monoclonal antibodies TRBP1/247 (Cedarlane Labs, Canada) [38], which was used at a dilution of 1:500, and the FITC-conjugated anti-mouse IgG (Sigma-Aldrich), which was used at a dilution of 1:400. Cells were analyzed on a fluorescence-activated cell sorter (Becton Dickinson & Co., Sunnyvale, CA).

For quantitative western blotting, an equal number (5×10^5^) of control and KIN-E RNAi cells were lysed, and cell lysate was fractionated on SDS-PAGE, transferred onto a PVDF membrane, and immunoblotted with anti-EP procyclin monoclonal antibody TRBP1/247 (1:1,000 dilution, Cedarlane Labs) and anti-TbPSA6 polyclonal antibody (1: 2,000 dilution), which detects the alpha-6 subunit of the 26S proteasome [39], for 1 hr at room temperature. After washing three times with TBST, the membrane was incubated with FITC-conjugated anti-mouse IgG (1: 400 dilution, Sigma-Aldrich) and IRDye 680LT anti-rabbit IgG (1:2,500 dilution, Li-Cor Cooperate), and western blot signals were captured using the Bio-Rad ChemiDoc MP imaging system, which allows multiplex fluorescent western blot imaging and quantitative analysis of protein bands.

### Co-immunoprecipitation and western blotting

Co-immunoprecipitation was carried out according to our previous procedures [40]. Briefly, cells (5x10^7^) were lysed by incubating with 1 ml immunoprecipitation buffer (25 mM Tris-HCl, pH7.6, 500 mM NaCl, 1 mM DTT, 1% NP-40, and protease inhibitor cocktail) for 30 min on ice. Cleared lysate was incubated with 50 μl settled IgG beads for 1 h at 4°C, and immunoprecipitates were washed six times with the immunoprecipitation buffer. Proteins bound to the IgG beads were eluted with 10% SDS, separated by SDS-PAGE, transferred onto a PVDF membrane, and immunoblotted with anti-HA antibody to detect 3HA-tagged KIN-E and its various mutants and with anti-Protein A antibody to detect PTP-tagged FLAM3. Cells expressing 3HA-tagged KIN-E alone and PTP-tagged FLAM3 alone were included as negative controls.

### *In vitro* GST pull-down

The DNA sequences encoding the importin-α-like domain (a.a. 413–684) and the m-calpain domain III-like domain (a.a. 713–997) of *KIN-E* gene were each cloned into the pGEX-4T-3 vector (Clontech). The resulting plasmids were transformed into *E*. *coli* BL21 strain. Expression of GST-fusion proteins was induced with 0.1 mM IPTG for 16 h at room temperature, and purified through glutathione sepharose beads. Purified GST fusion proteins bound on the beads were incubated at 4°C for 1 h with *T*. *brucei* lysate prepared by lysing *T*. *brucei* cells expressing PTP-FLAM3 in lysis buffer (25 mM Tris-HCl, pH7.6, 500 mM NaCl, 1 mM DTT, 1% NP-40, and protease inhibitor cocktail). The beads were washed six times with the lysis buffer, and bound proteins were eluted by boiling the beads in 1× SDS sampling buffer. Eluted proteins were separated on SDS-PAGE, transferred onto a PVDF membrane, and immunoblotted with anti-Protein A antibody to detect PTP-FLAM3. GST alone was used as the negative control. GST and GST-fusion proteins were stained by Coomassie Brilliant Blue dye.

### Preparation of cytoskeleton and western blotting

Cytoskeleton of *T*. *brucei* cells were prepared by incubating cells with PEME buffer (0.1 mM PEPES, pH6.9, 2 mM EGTA, 1 mM MgSO_4_, 0.1 mM EDTA) containing 1% Nonidet P-40 at room temperature for 5 min [41]. Cells were then spun down at 13,000 rpm in a microcentrifuge to separate the soluble cytosolic fraction and the insoluble cytoskeletal fraction. The cytoskeletal (pellet) fraction was re-suspended in PBS of the equal volume of the previously added PEME buffer. Both soluble and pellet fractions were boiled for 5 min after adding an equal volume of SDS-PAGE sampling buffer. Samples were separated by SDS-PAGE, transferred onto PVDF membrane, and immunoblotted with anti-Protein A (anti-ProtA) antibody to detect PTP-tagged FLAM3, anti-HA antibody to detect 3HA-tagged KIN-E and its mutants. The same blot was also probed with anti-α-tubulin mAb (Sigma-Aldrich) as cytoskeleton marker and with anti-TbPSA6 pAb as the cytosol marker.

### Immunofluorescence microscopy

Cells were washed once with PBS, adhered to the coverslips for 30 min at room temperature, fixed with cold methanol (-20°C) for 30 min, and then rehydrated with PBS for 10 min at room temperature. Cells adhered on the coverslips were blocked with 3% BSA in PBS for 1 h at room temperature, and incubated with the primary antibody for 1 h at room temperature. The following primary antibodies were used: FITC-conjugated anti-HA monoclonal antibody for 3HA-tagged proteins (1:400 dilution, Sigma-Aldrich), anti-Protein A polyclonal antibody for PTP-tagged proteins (1:400 dilution, Sigma-Aldrich), anti-CC2D polyclonal antibody (1:1,000 dilution) [42] for the FAZ filament [43], 20H5 monoclonal antibody (1:400 dilution) for the bilobe [44], anti-TbSAS-6 antibody (1:400 dilution) for basal body [45], YL 1/2 monoclonal antibody (1: 2,000 dilution) for mature basal body [46], L8C4 (anti-PFR2) monoclonal antibody (1:50 dilution) for the flagellum [43], L3B2 (anti-FAZ1) monoclonal antibody (1: 50 dilution) for the FAZ filament [43], and anti-CRAM polyclonal antibody (1:400 dilution) for the flagellar pocket [47]. Subsequently, cells were washed three times with PBS, and then incubated with FITC-conjugated anti-mouse IgG (1:400 dilution, Sigma-Aldrich) or Cy3-conjugated anti-rabbit IgG (1:400 dilution, Sigma-Aldrich) for 1 h at room temperature. Cells on the coverslips were washed three times with PBS, mounted with DAPI-containing VectaShield mounting medium (Vector Labs), and imaged under an inverted fluorescence microscope (Olympus IX71) equipped with a cooled CCD camera (model Orca-ER, Hamamatsu) and a PlanApo N 60x1.42-NA lens. Images were acquired using the Slidebook 5 software.

### Scanning electron microscopy

Scanning electron microscopy was performed essentially as described in our previous publications [40,48]. Cells were settled onto coverslips and fixed with 2.5% (v/v) glutaraldehyde in PBS for 2 hours at room temperature. Cells were washed three times with PBS, and then dehydrated in alcohol. After critical point drying, samples were coated with a 8-nm metal film (Pt:Pd 80:20, Ted Pella Inc.) using a sputter-coater (Cressington Sputter Coater 208 HR, Ted Pella Inc.), and then imaged using Nova NanoSEM 230 (FEI). The scanning work distance was at 5 mm, and the accelerating high voltage was at 8 kV.

### Statistical analysis

Statistical analysis was performed using the *t*-test in the Microsoft Excel software. Detailed *n* values for each panel in the figures were stated in the corresponding legends. For immunofluorescence microscopy, images were randomly taken and all cells in each image were counted.

## Supporting information

S1 Fig Knockdown of FLAM3 by RNAi in the procyclic form of *T*. *brucei*. (**A**). Western blotting to monitor the efficiency of FLAM3 RNAi. FLAM3 was endogenously tagged with an N-terminal PTP epitope in FLAM3 RNAi cell line, and was detected by anti-Protein A antibody. (**B**). FLAM3 RNAi caused a severe growth defect.
(PDF)
