# Supplementary figures and images for: An orphan kinesin controls trypanosome morphology transitions by targeting FLAM3 to the flagellum
Source: PLoS Pathog. 2018 May 29;14(5):e1007101. doi: 10.1371/journal.ppat.1007101 (PMC5993322; doi:10.1371/journal.ppat.1007101)

S1 Figure

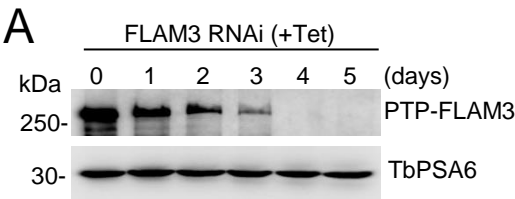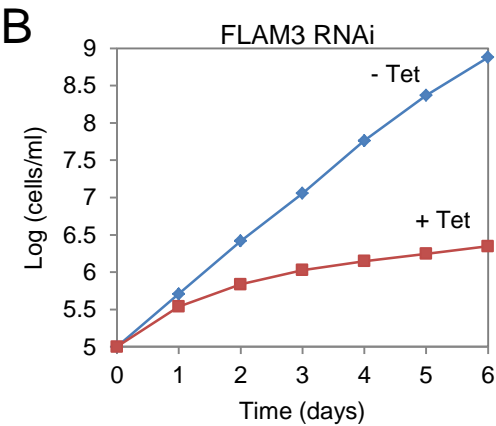

Supplement: S1 Fig — (A). Western blotting to monitor the efficiency of FLAM3 RNAi. FLAM3 was endogenously tagged with an N-terminal PTP epitope in FLAM3 RNAi cell line, and was detected by anti-Protein A antibody. (B). FLAM3 RNAi caused a severe growth defect. (PDF) [file ppat.1007101.s001.pdf]
